# Inhibitory Effect of Bovine Adipose-Derived Mesenchymal Stem Cells on Lipopolysaccharide Induced Inflammation of Endometrial Epithelial Cells in Dairy Cows

**DOI:** 10.3389/fvets.2021.726328

**Published:** 2021-10-21

**Authors:** Wengeng Lu, Zheng-Mei Xu, Qing Liu, Nan-Nan Yu, Jia-Bin Yu, Wei-Long Li, Ying-Ying Mao, Zhenzhen Du, Linqing Si, Siqi Yuan, Jidong Jin, Shixin Fu, Dongbo Sun, Ying-Hao Han

**Affiliations:** ^1^Laboratory of Theriogenology and Reproductive Health, College of Animal Science and Veterinary Medicine, Heilongjiang Bayi Agricultural University, Daqing, China; ^2^Heilongjiang Provincial Key Laboratory of Prevention and Control of Bovine Diseases, College of Animal Science and Veterinary Medicine, Heilongjiang Bayi Agricultural University, Daqing, China; ^3^Laboratory of Stem Cell Therapy and Regenration Biology, College of Life Science and Biotechnology, Heilongjiang Bayi Agricultural University, Daqing, China; ^4^Cofeed Feedmill (Changchun) Co., Ltd., Changchun, China

**Keywords:** bovine adipose-derived mesenchymal stem cell, inflammation, lipopolysaccharide, endometritis, dairy cows

## Abstract

Endometritis is a disease that affects reproductive health in dairy cows and causes serious economic damage to the dairy industry world-wide. Although in recent years, the application of mesenchymal stem cell (MSC) therapy for the treatment of inflammatory diseases has attracted much attention, there are few reports of the use of MSCs in dairy cows. In the present study, our objective was to explore the inhibitory effects of bovine adipose-derived mesenchymal stem cells (bAD-MSCs) on lipopolysaccharide (LPS) induced inflammation in bovine endometrial epithelial cells (bEECs) along with the potential underlying molecular mechanisms. We characterized isolated bAD-MSCs using cell surface marker staining and adipogenic/osteogenic differentiation, and analyzed them using immunofluorescence, flow cytometry (surface marker staining), and adipogenic and osteogenic differentiation. Furthermore, to understand the anti-inflammatory effects of bAD-MSCs on LPS induced bEEC inflammation, we used a bAD-MSC/bEEC co-culture system. The results showed that bAD-MSC treatments could significantly decrease LPS induced bEEC apoptosis and pro-inflammatory cytokine expression levels, such as interleukin-1β (IL-1β), interleukin-6 (IL-6), and tumor necrosis factor-alpha (TNF-α). Furthermore, our results showed that bAD-MSC treatments could also significantly downregulate LPS induced p38, IkB-a, and JAK1 phosphorylation and Bax protein expression levels, which are closely related to inflammatory progress and cellular apoptosis in bEECs. Our findings demonstrate that bAD-MSCs play an inhibitory role in LPS induced bEEC inflammation and provide new insights for the clinical therapy of endometritis in dairy cows.

## Introduction

In recent years, the incidence of uterine disease in dairy cows has shown an increasing trend, and has become one of the most important factors affecting their reproductive efficiency (1). The endometrium plays an essential role in embryo implantation, embryo development, and sperm motility (2). Endometritis is a type of refractory uterine disease, which is mainly caused by pathogenic microorganisms (such as *Escherichia coli* and *Streptococcus*) infecting the endometrium during or after parturition (3). Endometritis is one of the most common reasons for infertility in dairy cows and causes huge annual losses to the dairy industry. A survey of the literature shows that in the United States uterine inflammation can cause losses of around US$171.9 ± 47.88 in first lactation primiparous heifers, while this figure is around US$262.65 ± 56.15 per lactation in multiparous cows (4). Currently, the treatment for bovine endometritis includes injection with antibiotics and prostaglandin F2 alpha (PGF_2α_), and flushing of the uterus with iodine solution, but the cure rate is not satisfactory. Therefore, there is an urgent need to find new and effective treatments for endometritis in dairy cows.

Mesenchymal stem cells (MSCs) are a type of early undifferentiated adult stem cells that have the potential for self-renewal and multilineage differentiation. Compared with other stem cells (such as embryonic stem cell, germinal stem cell and neural stem cell), MSCs have a wide range of sources and can be obtained from bone marrow, adipose tissue, umbilical cords, and the placenta (5–7). MSCs can differentiate into adipocytes, chondrocytes, cardiomyocytes, nerve cells, hepatocytes, osteoblasts, and other cell types (8–13); MSCs also show characteristics of immune regulation, anti-inflammation, and inhibit malignant cell proliferation (14). With in-depth research, scholars are starting to realize that the paracrine effects of MSCs play an important role in disease treatment. MSCs secrete soluble bioactive factors to that inhibit apoptosis, promote angiogenesis, stimulate the differentiation of progenitor cells, and regulate immune response to promote the regeneration of injured tissues (15). Based on these characteristics, MSCs have been widely used in the treatment of bone, heart, kidney, lung, brain, and liver injuries (16–20). It has been reported that MSCs can regulate natural killer cells (NK cells), dendritic cells, T lymphocytes, and B lymphocytes, either through cell contact or cytokine secretion to modulate immune responses (21–23). In addition, paracrine factors [such as transforming growth factor-β (TGF-β), tumor necrosis factor-inducible gene 6 protein (TSG-6), indoleamine 2,3-dioxygenase (IDO), and prostaglandin-E2 (PGE2)] released by MSCs also have strong anti-inflammatory effects (24). Consequently, MSCs and/or MSC-based biological preparations are becoming novel therapeutic tools for solving issues of antibiotic residues and intractable diseases in livestock (25). Bone marrow is the most common source of MSCs, but sample collection is not easy; whereas MSCs from adipose tissue, namely adipose-derived MSCs (AD-MSCs) have become a research hotspot due to their abundance and convenience of sample collection.

Based on the above information, this study focused on the inhibitory effects of bAD-MSCs on LPS induced inflammation in bovine endometrial epithelial cells (bEECs) along with the underlying molecular mechanisms *in vitro*. It provides theoretical support for the application of bovine adipose-derived mesenchymal stem cells (bAD-MSCs) in the prevention and treatment of endometritis in dairy cows.

## Materials and Methods

### Isolation and Culture of bAD-MSCs

Bovine adipose tissue cells were isolated using 0.25% enzymatic digestion. bADs were collected under sterile conditions from the neck of a Holstein cow (age, 3–4 years; female; weight, 640 ± 30 kg) at a slaughterhouse (Daqing Longda Food Co., Ltd., China). Samples were washed three times with phosphate buffer saline (PBS) containing 1% (vol/vol) penicillin and streptomycin (penicillin 100 U/mL, streptomycin 100 μg/mL; Beijing Solarbio Science & Technology Co., Ltd., Beijing, China). The vascular, fascia, and connective tissues were discarded, and the adipose tissue was quickly homogenized and subsequently digested using 0.25% trypsinase (Beijing Solarbio Science & Technology Co., Ltd.) for 6 h at 37°C, and cells were filtered using a 70 μm cell strainer (Wuxi NEST Biotechnology Co., Ltd., China). Cells were cultured in Dulbecco's modified Eagle medium, nutrient mixture F-12 (DMEM/F-12, Thermo Fisher Scientific, Grand Island, NY, USA) containing 10% (vol/vol) fetal bovine serum (Zhejiang Tianhang Biotechnology Co., Ltd., China), and 1% (vol/vol) penicillin and streptomycin (penicillin 100 U/mL, streptomycin 100 μg/mL; Beijing Solarbio Science & Technology Co., Ltd.) at 37 °C in a humidified atmosphere of 5% CO_2_. Each medium was changed every 24 h. The cells were passaged when the confluence was 70–80%.

### Detection of the Surface Antigens and Multilineage Differentiation Potential of bAD-MSCs

It has been previously demonstrated that the most common lineage for MSCs is to differentiate down the osteogenic and adipogenic lines (26). The fourth passage of bAD-MSCs was obtained, and the cell density was adjusted to 1 × 10^5^ cells/3.5 cm culture dishes. The cells were cultured in DMEM/F-12 (Thermo Fisher Scientific, Grand Island, NY, USA) containing 10% (vol/vol) FBS, and 1% (vol/vol) penicillin and streptomycin (penicillin 100 U/mL, streptomycin 100 μg/mL; Beijing Solarbio Science & Technology Co., Ltd.) at 37°C in a humidified atmosphere of 5% CO_2_ in 3.5 cm culture dishes. For osteogenic differentiation and adipogenic differentiation, the cells were divided into two groups: a differentiation culture group and a control group. When the cells achieved a confluency of 80%, the differentiation culture groups were treated with osteogenic induction medium (Biowit BW12007) and adipogenic induction medium (Biowit BW12006), respectively. Each medium was changed every 48 h. After 21 d of culture, the media were discarded, the cells were washed with PBS (calcium and magnesium free) three times and fixed with 4% formaldehyde solution for 20 min. The fixation fluid was then discarded followed by three washes with PBS. In the osteogenic and adipogenic induction groups, Alizarin Red S (Beijing Solarbio Science & Technology Co., Ltd.) and Oil Red O (Beijing Solarbio Science & Technology Co., Ltd.) were added for staining for 5 and 15 min, respectively, the staining solution was discarded, and the cells were washed three times with PBS. Finally, after mounting with neutral resin, the samples were observed under a microscope.

The bAD-MSCs from passage 4 were used for all assays, phenotypic analysis took place by immunofluorescence, flow cytometry, and differentiation culture. For immunofluorescence assays, cells were placed in 24-well plates at an initial density of 4 × 10^4^ with DMEM/F-12 supplemented with 10% fetal bovine serum (FBS; Zhejiang Tianhang Biotechnology Co., Ltd.) culture media, in a 37°C incubator with 5% CO_2_. After 24 h, each culture medium was removed and cells were washed three times with PBS; the cells were fixed with 4% formaldehyde in the dark for 10 min at room temperature (RT), and washed with PBS a further three times, each time for 5 min. Cells were then permeabilized with a PBS dilution of 0.1% Triton X-100 (T9284; Sigma-Aldrich, St. Louis, MO, USA) for 10 min, followed by a further three washes with PBS. The cells were then blocked with bovine serum albumin (BSA, Sigma-Aldrich) for 30 min. Subsequently, cells were incubated with primary antibodies CD73 (Proteintech; 1:200), CD44 (Biolegend; 1:200), CD45 (Biolegend; 1:200), and CD34 (Biolegend; 1:200) at 4°C overnight, followed by three washes with PBS. Different from the treatment methods of CD44, CD45 and CD34, the detection of the CD73 surface marker needed a secondary antibody [goat anti-mouse IgG conjugated with Cy3 (Beyotime Institute of Biotechnology, Shanghai, China)] incubation for 2 h. Cells were then stained with 4', 6-diamidino-2-phenylindole (DAPI, 1 μg/mL; Sigma-Aldrich) for 10 min. Subsequently, cells were visualized with a laser scanning confocal microscope (TCS SP5, Leica Microsystems, Wetzlar, Germany), and images were taken under an Olympus (Tokyo, Japan) FLUOVIEW FV1000 microscope.

Single-cell suspensions were prepared using enzymatic dissociation with trypsin-EDTA solution, fixed with 70% ethanol, and permeabilized with 0.05% Triton X-100 (Sigma- Aldrich) for 1.5 min. Subsequently, cells were centrifuged at 4°C for 3 min and blocked with BSA for 30 min. Cells were then incubated with the primary antibodies CD73 (Proteintech; 1:200), CD44 (Biolegend; 1:200), CD45 (Biolegend; 1:200), and CD34 (Biolegend; 1:200) at 4°C overnight, followed by three washes with PBS. CD73 was incubated with Cy3 conjugated goat anti-rabbit IgG (Beyotime Institute of Biotechnology, Shanghai, China; 1:200) at RT for 1 h. The samples were centrifuged at 4°C for 3 min and suspended in PBS. Lastly, cells were detected using flow cytometry (FACSCalibur, BD Biosciences, Franklin Lakes, NJ, USA) and results were analyzed using WinMDI (v.2.9, BD Biosciences) software.

### Establishment of a Bovine Endometritis Model Induced by LPS *in vitro*

A bovine endometrial epithelial cell (bEEC) line was established in our laboratory. Briefly, the caruncle tissue was cut into small sections (3–5 mm^2^), which were washed three times with phosphate-buffered saline (PBS; pH 7.3). Roswell Park Memorial Institute-1640 medium (RPMI-1640; HyClone, Beijing, China) containing 10% fetal calf serum (FCS; Sijiqing Biological Engineering Materials Co., Ltd., Hangzhou, Zhejiang, China) and 1% penicillin/streptomycin (10,000 IU/10,000 μg/ml; Beijing Solarbio Science & Technology Co., Ltd., Beijing, China) were added to the tissue sections attached to the cell culture dish (60 × 15 mm, NEST, China) and then incubated in an atmosphere of 5% CO2/95% air at 37°C. After 6 h, 1.5 ml of RPMI-640 was added to the bottom of the cell culture dish. Epithelioid cells were separated into two populations based on enzyme sensitivity. In order to identify the purity of endo epithelial cells, immunofluorescence was performed with cultures passaged three times using a primary antibody against cytokeratin 18 (CK 18; Abcam, Cambridge, UK) as described previously (27). The bEECs were removed from the liquid nitrogen tank for resuscitation culture and passage. They were then plated in 24-well plates at a density of 4 × 10^4^ cells/well in RPMI-1640 standard complete medium (Hyclone, Logan, UT, USA) containing 10% (vol/vol) FBS, and 1% (vol/vol) penicillin and streptomycin (penicillin 100 U/mL, streptomycin 100 μg/mL) at 37°C in a humidified atmosphere of 5% CO_2_. After bEEC adhesion (about 4 h), LPS (Sigma-Aldrich) at concentrations of free, 0.1, 1, and 10 μg/mL were added into different wells for 6 h to screen the best stimulation concentration. Then, after determining the optimal concentration, the optimal concentration (0.1ug/mL) is used to stimulate different times (0, 3, 6, 12, and 24 h) to screen the optimal stimulation time. Finally, the expression of inflammatory related genes (e.g., IL-1 β, IL-6, and TNF- α) were detected by real-time quantitative PCR. The same genes expressed at each concentration and each time point were analyzed to determine the optimal treatments.

### Co-culture Assays

The bAD-MSCs and bEECs were co-cultured in transwell plates with a pore size of 0.4 μm (3413, Corning, NY, USA) at ratios of 0.5:1 and 1:1, in the upper and lower compartments, respectively. Briefly, bAD-MSCs were resuscitated from frozen storage. The density of bAD-MSCs was adjusted to 3 × 10^4^ cells/well and 6 × 10^4^ cells/well. The cells were seeded into transwell chambers and placed in an incubator at 37°C and 5% CO_2_ for use. The bEECs (6 × 10^4^ cells/well) were then plated in 24-well plates and cultured for 4 h, and LPS at a concentration of 0.1 μg/mL (deemed the ideal concentration following experiment 2.3 above) was added after most of the cells had adhered to the plate wall. After stimulation with LPS for 6 h, all the original culture medium was discarded and 500 μL of DMEM/F-12 medium was supplemented. The transwell chambers were transferred into 24-well plates for co-culture for 48 h. After co-culture, each culture medium was discarded and the cells were washed with PBS, and then stained using an Annexin V-PE kit (Beijing Solarbio Science & Technology Co., Ltd.) to detect apoptosis. The stained cells were observed under a fluorescence microscope after incubation in accordance with the manufacturer's instructions. At the same time, the bEECs were collected and total RNA was extracted to detect the expression of IL-1 β, IL-6, and TNF-α. The experimental group was co-cultured after LPS stimulation. The LPS stimulated, but not the co-cultured, bEECs were set aside as the positive control group and bEECs (no LPS stimulation) were set as the negative control group.

### Detection of RNA Expression of Cytokines

Genes were detected using Quantitative Reverse-Transcription PCR (qRT-PCR) as described by Sun et al. (28). Trizol was used to extract total RNA from bEECs. Then, an ultraviolet visible spectrophotometer (UV-Vis; Thermo Fisher Scientific) was used to measure RNA purity. The optical density (OD)260/(OD)280 ratio of the total RNA was determined to be 1.9, and the intensity of the 28S ribosomal RNA band was about twice the intensity of the 18S ribosomal RNA band in total RNA samples; this indicated that the total RNA was of high quality. The cDNA was synthesized from total RNA using a PrimeScript RT Reagent Kit (Takara Biotechnology Co., Ltd., Dalian, China). The gene expression specific sequences of cows are shown in Table 1 (the primer sequences were generated as described previously) (29, 30). Subsequently, cDNA was synthesized using a reverse transcription kit (Takara Biotechnology Co., Ltd.). Total cDNA was used as the starting material for real-time PCR with FastStart Universal SYBR Green Master Mix (Roche Applied Science, Germany). Finally, the 2^−ΔΔCt^ method was used to analyze expression levels. All reactions were run in triplicate.

**Table 1:** Primer sequence of inflammatory related factor.

**Gene**	**Primer**	**Sequence**
β-Actin	Forward	CCAAGGCCAACCGTGAGAAAAT
	Reverse	CCACATTCCGTGAGGATCTTCA
IL-1β	Forward	ATGAAGAGCTGCATCCAACA
	Reverse	ATGGAAGACATGTGCGTAGG
TNF-α	Forward	TGACGGGCTTTACCTCATCT
	Reverse	TGATGGCAGACAGGATGTTG
Il-6	Forward	AACGAGTGGGTAAAGAACGC
	Reverse	CTGACCAGAGGAGGGAATGC

### Protein Extraction and Western Blotting Analysis

To verify bAD-MSCs mediated processes, we extracted the total protein from endometrial cells and performed SDS-PAGE; the immunoreactive proteins visualized were IκB-α, P-JAK1, P-p38, p-38, and Bax. bAD-MSCs were seeded into transwell chambers at a density of 6 × 10^4^ cells per chambers and placed in an incubator at 37°C and 5% CO_2_ for use. bEECs were seeded onto 24-well plates at a density of 6 × 10^4^ cells per well for 4 h to adhere and then treated with LPS for 6 h. Then, bAD-MSCs and bEECs were co-cultured in transwell plates in the upper and lower compartments, respectively. Cells were cultured in DMEM/F-12 medium supplemented with 10% FBS in an incubator at 37°C and 5% CO_2_ for 12, 24, and 48 h, and then cells were harvested and added to the lysate. Lysates were then centrifuged at 13,201 × g for 30 min at 4°C and collected for further analysis. The protein concentration was determined using the Bradford protein assay. The absorbance of the mixture at 595 nm was determined using a UV-Visible spectrophotometer (Thermo Fisher Scientific). Total proteins were extracted from endometrial cells and SDS-PAGE was performed. Briefly, equal amounts of protein (25 μg) were separated on an 8–15% Tris-glycine gel and electro transferred onto polyvinylidene difluoride (PVDF) membranes. The PVDF membranes were blocked with 5% non-fat dried milk, and membranes were incubated overnight at 4°C with primary antibodies against β-actin (1:1,000, Sigma-Aldrich), IκB-α (1:1,000, Bioss biology, Beijing, China), P-JAK1 (1:2,000, Santa Cruz, CA, USA), P-p38 (1:1,000, Sigma-Aldrich), p-38 (1:1,000, Bioss Biology), and Bax (1:1,000, Bioss Biology). These were followed by horse-radish peroxidase (HRP)-conjugated anti-rabbit or anti-mouse IgG (Thermo Fisher Scientific) at RT for 1 h. Target protein bands were revealed with an enhanced chemiluminescence (ECL) detection kit (Thermo Fisher Scientific), and the images were analyzed using Image-Pro Plus 6.0 software.

### Statistical Analysis

All experiments and determinations were repeated three times. Data were analyzed using IBM SPSS Statistics v.20.0 and were tested for normality. The first, data of mRNA expression of IL-1β, IL-6, TNF-α and the protein expression of IκB-α, pJAK-1, Bax, pP38 were analyzed through Excel. And then differences of IL-1β, IL-6, TNF-α between control group and LPS group and differences of IκB-α, pJAK-1, Bax, pP38 between LPS group and co-culture group were compared using one-way ANOVA followed by Bonferroni correction. All values for variables are expressed as the mean and standard error of the mean (mean ± SEM). *P* < 0.05 were considered to be significant; when <0.01, they were considered to be highly significant.

## Results

### Isolation and Characterization of bAD-MSCs

**Figure 1:**
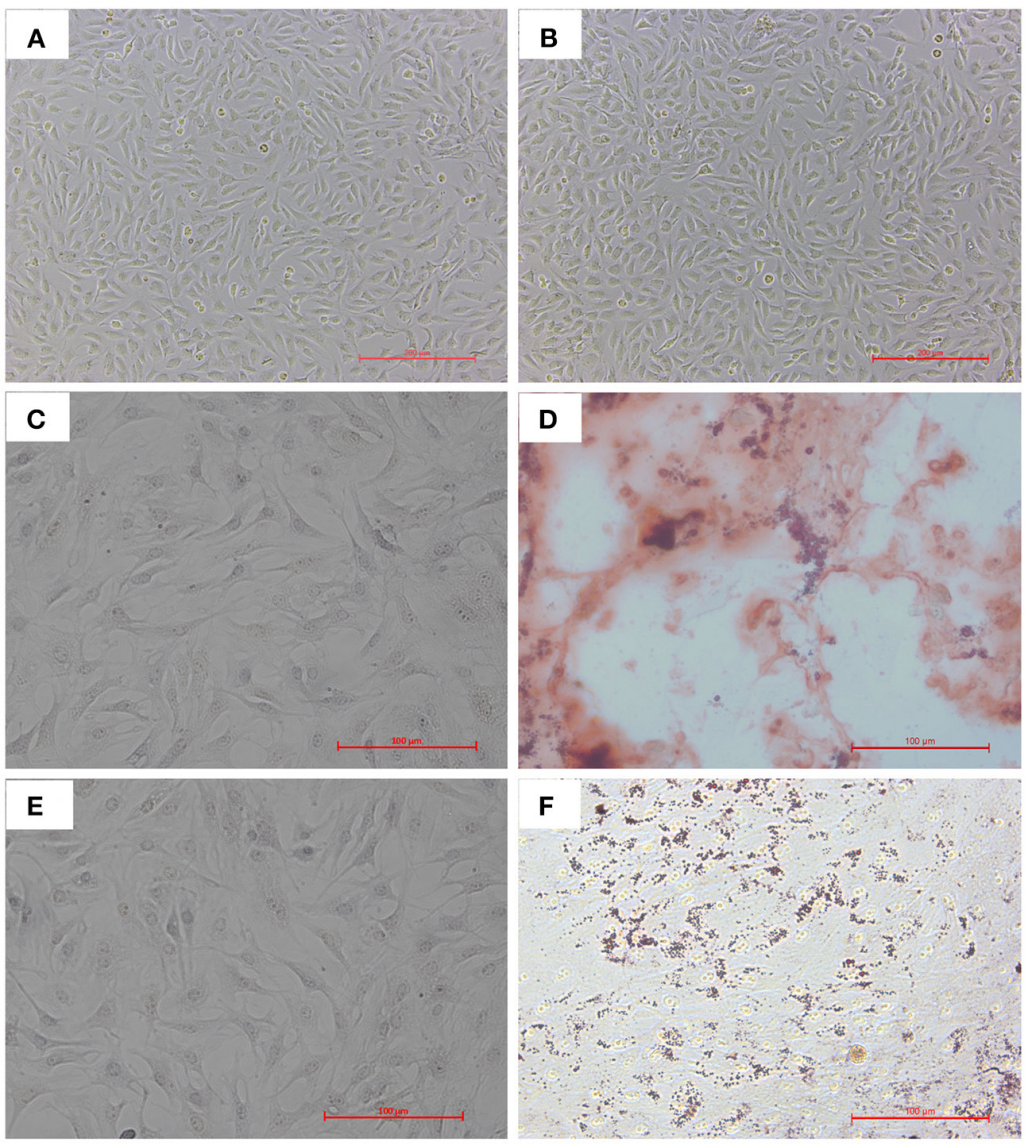
Cultivation and differentiation of bAD-MSCs. **(A,B)** Cell morphology of different parts at P0 and P4. **(C,D)** Osteogenic differentiation after 21 d showing calcium deposits stained with Alizarin Red S (C is control). **(E,F)** Adipogenic differentiation after 21 d showing lipid droplets stained with Oil Red O (E is control) (Scale bar = 200 μm).

**Figure 2:**
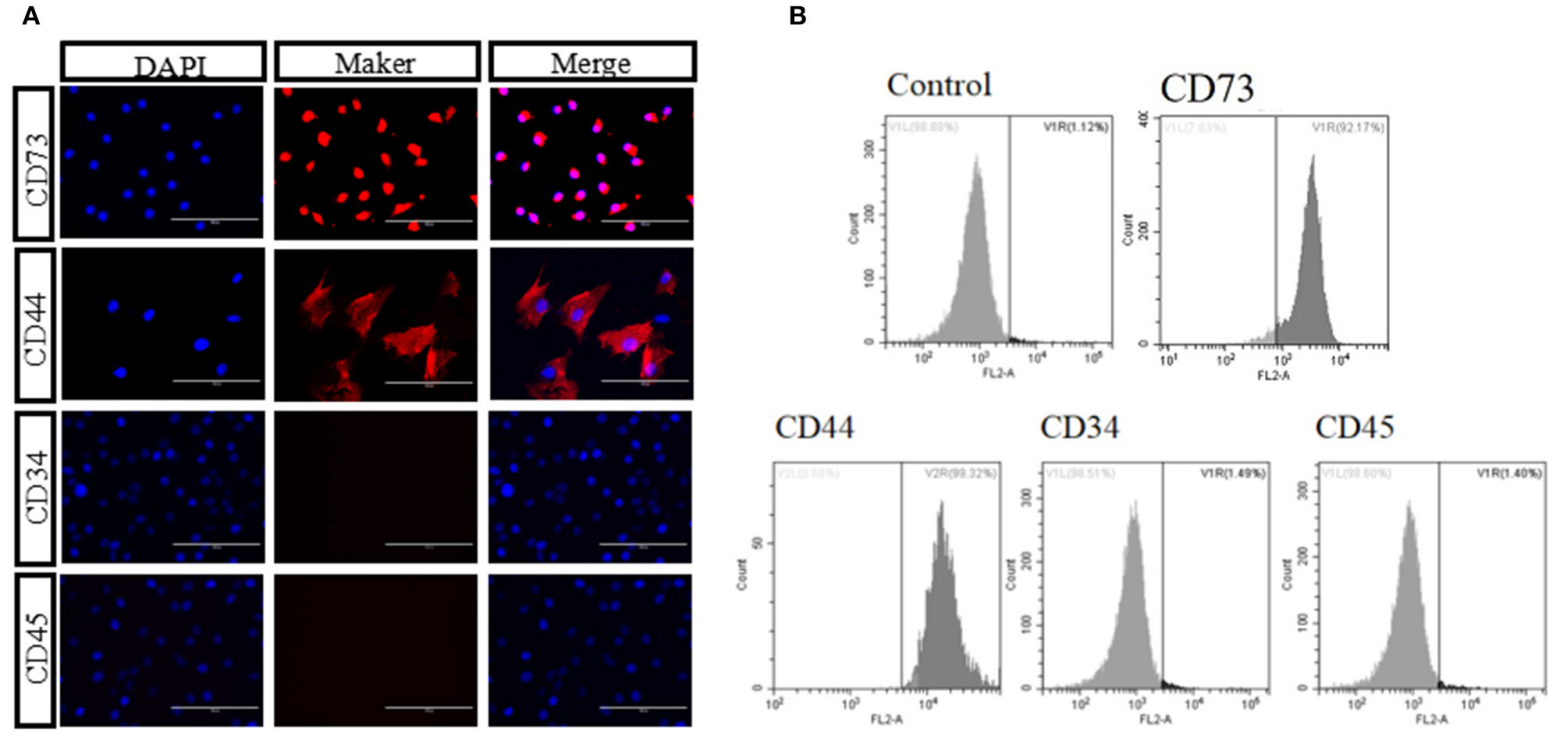
Identification of bAD-MSCs. **(A,B)** Identification marker CD44, CD73, CD45, and CD34 using immunofluorescence and flow cytometry (Scale bar = 100 μm).

We isolated and cultured bAD-MSCs as described in the Methods section. *In vitro* observation showed that cells were spindle-shaped and exhibited fibroblast-like adherent growth (Figures 1A,B). Furthermore, we cultured bAD-MSCs in differentiation medium and found that the isolated bAD-MSCs were able to differentiate into osteoblasts (Figures 1C,D) and adipocytes (Figures 1E,F), as demonstrated by Alizarin Red and Oil Red O staining; thus indicating the successful extraction of bAD-MSCs. The results of immunofluorescence microscopy and flow cytometry assays showed that isolated cells were strongly stained with CD73 and CD44 antibodies, the positive surface markers for bAD-MSCs, but showed a low binding affinity to CD45 and CD34 (negative surface markers for bAD-MSCs; Figures 2A,B).

### Effect of Different Concentrations of LPS on Pro-inflammatory Cytokine Relate Gene Expression in bEECs

**Figure 3:**
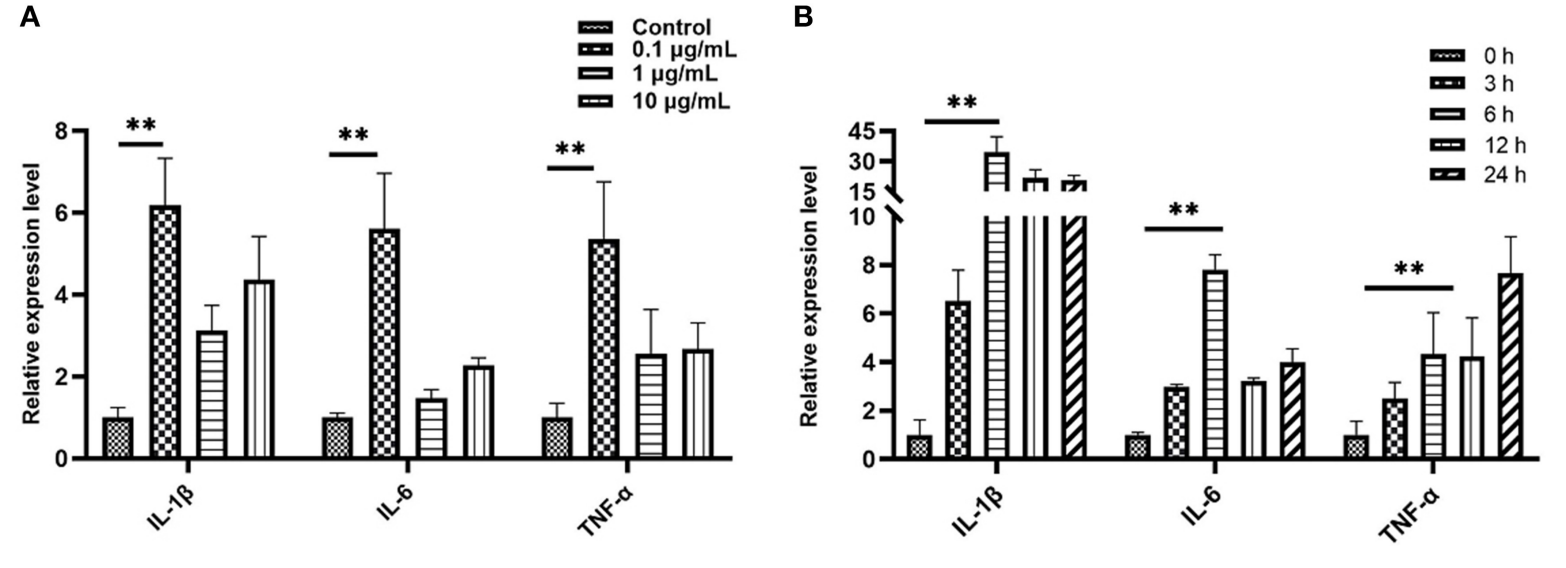
Quantitative PCR analysis for the expression of pro-inflammatory cytokines such as IL-1β, IL-6, and TNF-α. **(A)** Screening stimulation optimum concentration of LPS (stimulation for 6 h). **(B)** Screening stimulation optimum timing with 0.1 μg/mL. Values are mean ± SD (*n* = 4). Significance is indicated by **P < 0.01.

We treated bEECs with various concentrations of LPS (free, 0.1, 1, and10 μg/mL) and analyzed pro-inflammatory gene expression levels using qRT- PCR. In the screening of LPS stimulation concentration we found that, compared with the control group (LPS free), the relative expression levels of IL-1β, IL-6, and TNF-α were 6.19, 5.61, and 5.37, respectively, and were significantly increased in the 0.1μg/mL treatment of the LPS group (*P* < 0.01; Figure 3A). In the screening of LPS (0.1 μg/mL) stimulation time, when compared with the control group (0 h = none), the relative expression levels of IL-1β, IL-6, and TNF-α were 34.65, 7.81, and 4.33, respectively, and were increased highly significantly in the 6 h group (*P* < 0.01; Figure 3B). The data were processed and analyzed using Graphpad.

### bAD-MSC Treatment Downregulates Pro-inflammatory Cytokine Expression in LPS Induced bEECs

**Figure 4:**
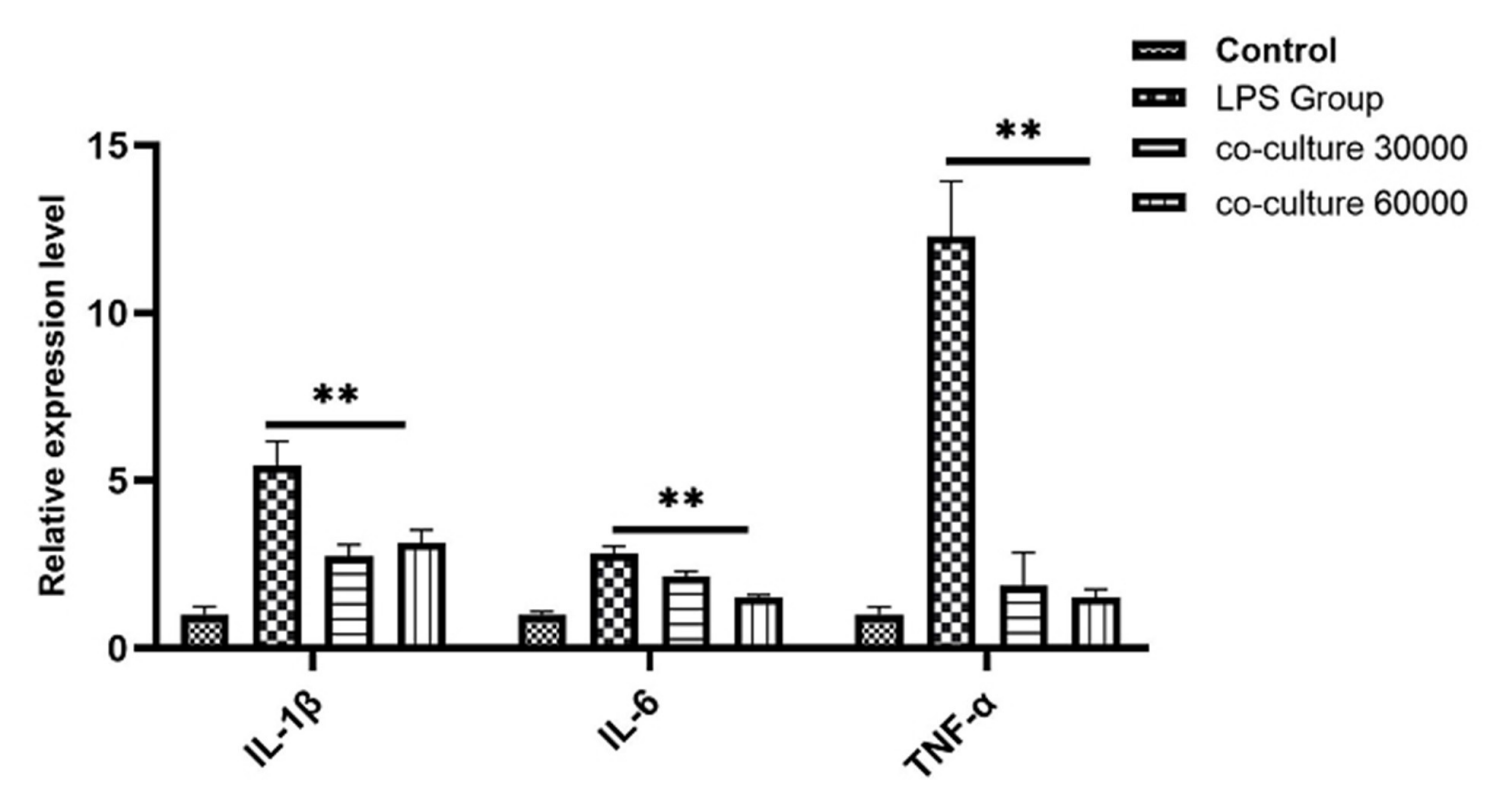
Quantitative PCR analysis for the expression of pro-inflammatory markers such as IL-1β, IL-6, and TNF-α when co-cultured with bAD-MSC 30,000 cells and 60,000 cells in the presence of 0.1 μg/mL of LPSs. Expression levels normalized to the reference gene β-actin. Values are mean ± SD (*n* = 4). Significance is indicated by **P < 0.01.

To investigate the anti-inflammatory effect of bAD-MSCs in the *in vitro* endometritis model, we seeded bAD-MSCs and bEECs into the transwell co-culture system with the indicated number of cells, followed by LPS stimulation. As shown in Figure 4, the relative expression levels of IL-1β, IL-6, and TNF-α were 5.45, 2.82, and 12.28 in LPS stimulated group, respectively, while the relative expression levels of IL-1β, IL-6, and TNF-α were 3.14, 1.53, and 1.52 in co-culture 60,000 group, respectively. The bAD-MSCs treatments highly significantly decreased the LPS induced pro-inflammatory cytokine expression levels, including IL-1β, IL-6, and TNF-α, in the bEECs (*P* < 0.01). This result demonstrated that the paracrine factors of bAD-MSCs had an anti-inflammatory effect in the endometrial epithelial cell inflammatory model.

### bAD-MSCs Downregulate Iκb-α and p-JAK1 Protein Expression in LPS Induced bEECs

**Figure 5:**
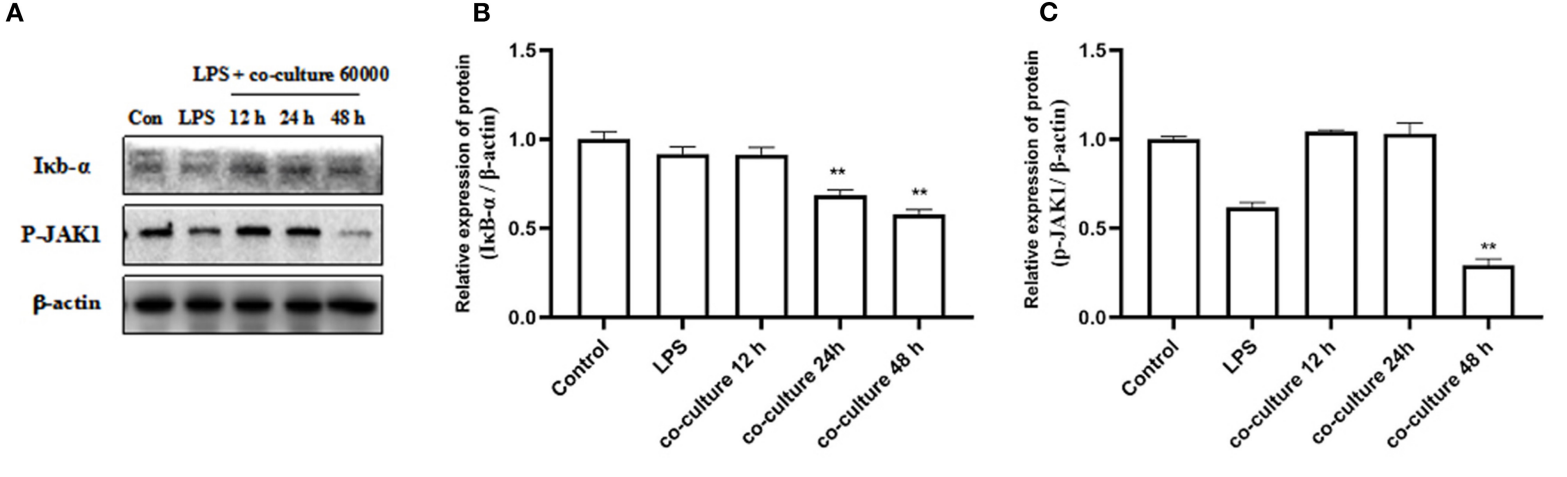
Expression of inflammation related proteins. bEECs were treated with 0.1 μg/mL LPSs for 6 h, co-cultured with bAD-MSCs 60 000 cells for 12, 24, and 48 h. **(A)** Western blot analysis of IκB-α and P-JAK1 proteins. **(B)** Relative expression levels of IκB-α protein. **(C)** Relative expression levels of P-JAK1 protein. Significance is indicated by **P < 0.01.

To understand the possible molecular mechanisms for the inhibitory effects of bAD-MSCs on LPS induced pro-inflammatory cytokine expression in bEECs, we examined the activation level of the Iκb-α and p-JAK1 signaling pathways in bAD-MSC/bEEC co-culture systems after LPS stimulation. As shown in Figure 5, treatment with bAD-MSCs significantly downregulated LPS induced Iκb-α protein expression (relative expression of Iκb-α protein were 0.92 and 0.58 in LPS group and in co-culture 48 h, respectively) and p-JAK1 phosphorylation in bEECs (relative expression of p-JAK1 phosphorylation protein were 0.62 and 0.29 in LPS group and in co-culture 48 h, respectively)(*P* < 0.01).

### bAD-MSCs Inhibit LPS Induced bEEC Apoptosis

**Figure 6:**
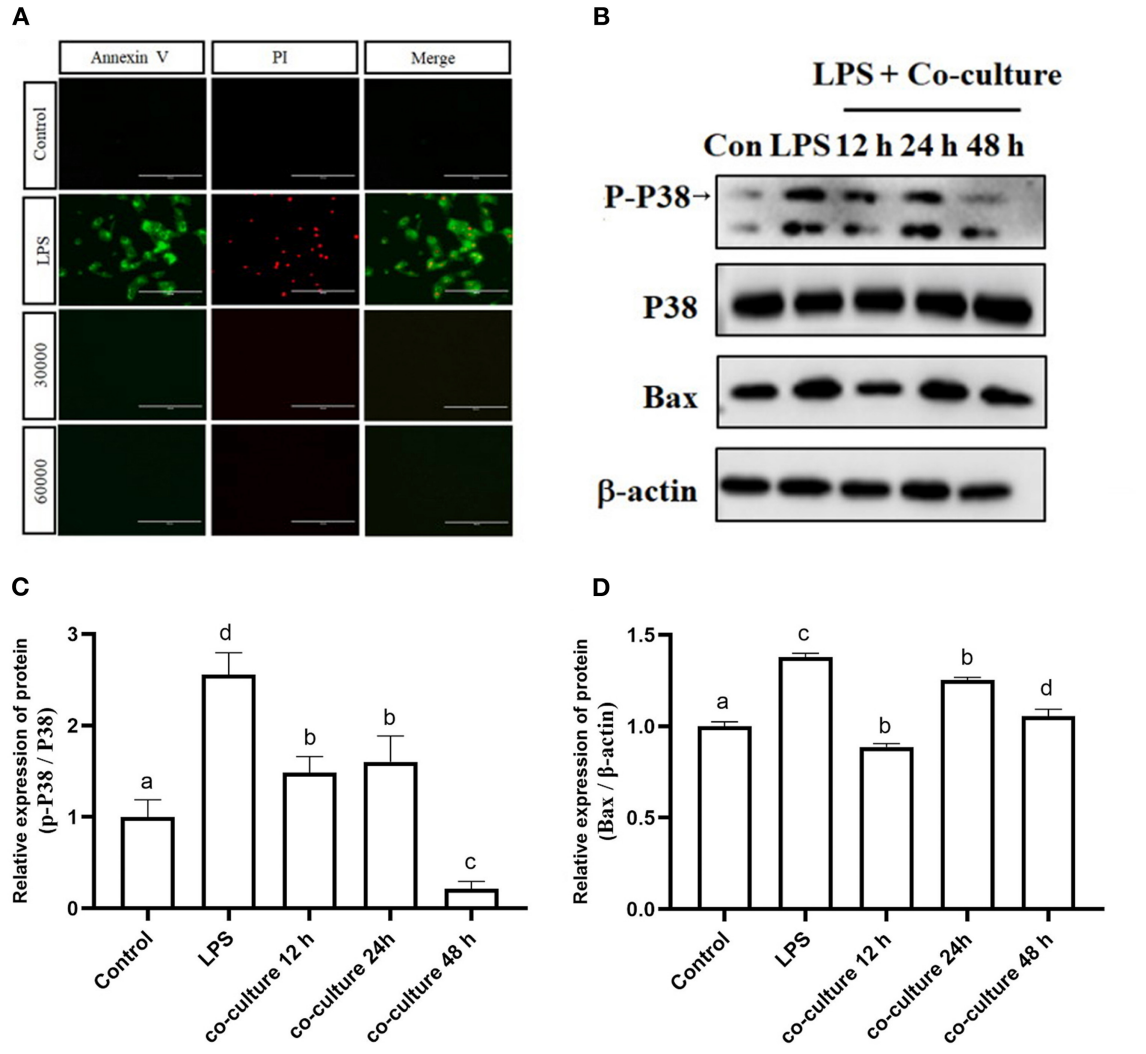
Effects of bAD-MSCs on LPS induced apoptosis of bEECs. **(A)** Immunofluorescence reactions for bEEC apoptosis by Annexin V-FITC and PI. bEECs were treated with 0.1 μg/mL LPSs for 6 h, co-cultured with bAD-MSCs 30,000 cells, and 60,000 cells for 48 h. The first column shows Annexin V by FITC, while the second column shows cell nuclei labeled with PI. The last column shows merged images with FITC and PI. (Scale bar = 100 μm). **(B)** Western blot analysis of p38, P-p38, and Bax proteins. bEECs were treated with 0.1 μg/mL LPSs for 6 h, and co-cultured with bAD-MSCs for 12, 24, and 48 h. **(C)** Relative protein expression levels of P-p38/p38. **(D)** Relative expression levels of Bax protein. Different lowercase letters in bar charts indicate significant differences (*P* < 0.05).

To verify the effect of bAD-MSCs on LPS induced apoptosis in bEECs, we observed the cellular apoptosis levels of bEECs after LPS stimulation in a bAD-MSC/bEEC co-culture system. According to microscopic examination, we found that the bAD-MSC treatment significantly decreased LPS induced apoptosis in bEECs stained with an Annexin-V-FITC/PI detection kit (Figure 6A). Furthermore, to understand the possible mechanisms of the anti-apoptotic effects of bAD-MSCs on LPS induced bEECs, we also examined the p38 signaling pathway and Bax (pro-apoptotic protein) protein expression levels. As shown in Figures 6B–D, the bAD-MSC treatment significantly downregulated LPS induced p38 phosphorylation (P-p38) level (relative expression levels were 2.56 and 0.22 in LPS group and in co-culture 48 h group, respectively, *P* < 0.01) as well as Bax protein expression (relative expression levels were 1.38 and 1.06 in LPS group and in co-culture 48 h group, respectively, *P* < 0.05) in bAD-MSC/bEEC co-culture systems. These results suggest that bAD-MSCs can inhibit LPS induced bEEC apoptosis.

## Discussion

MSCs are adult stem cells that originate from the mesoderm. MSCs have been successfully isolated and cultured from the skin, tooth tissue, peripheral blood, the dermis, and adipose tissue (31–33). They were initially found in bone marrow, but studies have shown that MSCs constitute between 0.001 and 0.01% of the total number of bone marrow cells, while in adipose tissue this figure can be as high as 2% of nucleated cells (34, 35). Therefore, adipose tissue can be an excellent source of MSCs. Recently, researchers have paid close attention to the therapeutic performance of MSCs. MSCs used in cell therapy need to satisfy the following three conditions: in the process of standard culture: they must have the characteristics of adherent growth; express positive markers, such as CD105, CD90, CD44, CD73, instead of negative markers, such as CD34, CD45, CD19; and can exhibit osteogenic and adipogenic differentiation *in vitro* (36). In the present study, we found that the isolated bAD-MSCs from bovine adipose tissues were not only strongly positive to the CD73 and CD44 antibodies, while negative to the antibodies for CD45 and CD34, but also exhibited osteogenic and adipogenic differentiation, which indicated that the cells we isolated and cultured had the characteristics of MSCs and could be used in the further study.

Through their ability to secrete endogenous regulatory factors of inflammation, MSCs have an anti-inflammatory effect, which can affect the progression of various inflammatory diseases through immunomodulation (37). For example, IL-10 (Interleukin-10), the activated IL-10 receptor complex can activate Janus kinase (JAK) and cause the phosphorylation of transcription (STAT), thus affecting the transcription of target genes and playing the anti-inflammatory regulatory role (38, 39). Likewise, IDO (Indoleamine 2,3-dioxygenase), a study shows that IFN-γ production by activated T-cells is correlated with the induction of IDO expression in MSCs via the IFN-γ-JAK-STAT1 pathway, thereby inhibiting T-cell proliferation (40). MSCs have a high degree of plasticity and the inherent ability to perceive changes in the microenvironment, so the different immunoregulatory effects of MSCs depend on their environment (41). In the process of inflammation, MSCs can inhibit archetypical inflammatory behaviors of target cells and promote anti-inflammatory, pro-apoptosis, and/or the generation of regulatory cells (42, 43). Studies have shown that MSCs interact with vascular endothelial cells to regulate inflammatory infiltration (44). On the other hand, bone marrow mesenchymal stem cells act through the release of soluble factors, so there are still many unknown areas to explore in cell therapy using mesenchymal stem cells (45).

Endometritis is an obstetric disease, which seriously reduces the reproductive performance of cattle. The etiology of endometritis is very complex; it is mostly caused by fungal, bacterial, and parasitic infections (46, 47). LPSs are one of the important causes of endometritis in dairy cows; they can induce an inflammatory response by stimulating bEECs to secrete inflammatory cytokines (48). Therefore, we chose LPSs to establish an *in vitro* bovine endometritis model to study the anti-inflammatory effect of MSCs. The expression of IL-1β, IL-6, TNF-α, and other inflammatory genes was increased after LPS stimulation. The results showed that LPSs successfully induced a cellular immune response by activating IL-1β and TNF-α secretion pathways. At the same time overexpression of IL-1β and TNF-α often leads to the recruitment of macrophages and activation of inflammatory cytokine interleukin-6 (IL-6) (49). In this experiment, bAD-MSCs were co-cultured within a bovine endometrial epithelial cell inflammation model; we found that the levels of IL-1β, TNF-α, and IL-6 were significantly lower than those in the endometritis model group. These results suggest that bAD-MSCs have a certain immunosuppressive capacity and can play an anti-inflammatory role. Therefore, we went on to detect the expression of inflammatory related proteins. Inflammation is a complex process when tissue cells are infected or damaged, such as during LPS stimulation. When stimulated by LPSs, the TLR4 pathway was activated, NF-κB was transferred to the nucleus, IκB-α was induced, and other proteins were expressed. Janus kinases (JAKs) are important signaling mediators downstream of many pro-inflammatory cytokines. Small molecule inhibitors of JAKs have been used as therapeutic agents for the treatment of inflammation, in which the Jak1/STAT3 signal is involved in the pathogenesis of inflammatory diseases (50, 51). Cytokine receptors use the Janus kinase signal transduction and transcription activation (JAK-STAT) pathway for signal transduction (52). When the cytokines bind to their cognate receptors, this activates the receptor-associated JAK (53). In our study, the expression of IκB-α and P-JAK1 proteins in endometrial epithelial cells of dairy cows was significantly increased when stimulated by LPSs. However, the expression of IκB-α and p-JAK1 were decreased when co-cultured with bAD-MSCs. This result may be related to some immunoregulatory characteristics of MSCs (such as TGF-β, IL-10, PGE_2_, TSG-6), and we will continue to investigate this issue. These outcomes suggest that bAD-MSCs play an anti-inflammatory role via the regulation of the IκB-α and JAK1 signaling pathways activated by LPSs. This is the first study of the role of the JAK1 signaling pathway in the treatment of endometritis by bAD-MSCs, and the results of this study offer a new therapeutic target for the treatment of bovine endometritis.

LPS induced inflammatory damage usually leads to changes in cell membrane permeability and the expression of apoptosis related proteins (54). We found that co-culture with bAD-MSCs can also reduce bEEC apoptosis. In the current experiment, the apoptosis of bEECs was induced by LPSs. Under an inverted fluorescence microscope, phosphatidylserine, and nuclei were stained by Annexin V-FITC and PI. However, the staining of Annexin V-FITC and PI in bEECs decreased significantly after co-culture with bAD-MSCs. To this point in the study, we had made a preliminarily elucidation of the anti-apoptotic ability of bAD-MSCs. We then detected changes in apoptosis related proteins when LPSs stimulated bAD-MSCs. The P38 MAPK signaling pathway is a recognized stress response pathway. After being stimulated and activated, p38 MAPK regulates cell apoptosis and proliferation through various means. Studies have shown that p38 MAPK can regulate the synthesis of Bax protein and then mediate cell apoptosis (55). When bEECs treated with LPSs were co-cultured with bAD-MSCs, the expression of P-p38, p38, and Bax protein decreased significantly. From this we can infer that bAD-MSCs can inhibit the p38 signaling pathway activated by LPSs, thus reducing the pro-apoptotic protein Bax and playing an anti-apoptotic role. However, it is still not clear how bAD-MSCs regulate the changes of these signaling pathways. According to the existing literature, we speculate that it may be regulated by the release of paracrine factors and/or exosomes; this theory requires further study in the future.

## Conclusions

In conclusion, in this paper, we studied bAD-MSC therapy of endometritis using a bovine *in vitro* model. The results shed further light on the molecular mechanisms underlying the suggestion that bAD-MSCs can inhibit LPS induced bEEC inflammatory response and apoptosis by downregulating p38, IkB-a, and JAK1 phosphorylation and Bax protein expression levels. This study provides a theoretical basis for the future application of MSC therapy in the prevention and treatment of endometritis in dairy cows.

## Data Availability Statement

The original contributions presented in the study are included in the article/supplementary material, further inquiries can be directed to the corresponding authors.

## Author Contributions

WL, Z-MX, and Y-HH conceived the study and wrote the manuscript. WL, Z-MX, QL, N-NY, J-BY, W-LL, Y-YM, ZD, LS, SY, JJ, SF, and DS carried out experiments and data analysis. WL, DS, and Y-HH interpreted the data. All authors approved the final version.

## Funding

This work was supported by Heilongjiang Provincial Natural Science Foundation of China (Grant No. LH2020C084), the Key Scientific Research Program of the General Bureau of State Farms of Heilongjiang Proviance (Grant No: HKKY190302), and Three longitudinal and three transverse scientific research talent support programs-Basic cultivation plan projects of Heilongjiang Bayi Agricultural University (Grant No. ZRCPY201808).

## Conflict of Interest

JJ is employed by Cofeed Feedmill (Changchun) Co., Ltd., Changchun, Jilin Province, China. The remaining authors declare that the research was conducted in the absence of any commercial or financial relationships that could be construed as a potential conflict of interest.

## Publisher's Note

All claims expressed in this article are solely those of the authors and do not necessarily represent those of their affiliated organizations, or those of the publisher, the editors and the reviewers. Any product that may be evaluated in this article, or claim that may be made by its manufacturer, is not guaranteed or endorsed by the publisher.
